# *Where There’s Hope, There’s Life* ^1^: On the Importance of Hope in Health Care

**DOI:** 10.1093/jmp/jhae037

**Published:** 2024-11-01

**Authors:** Steve Clarke, Justin Oakley

**Affiliations:** Charles Sturt University, Wagga Wagga, New South Wales, Australia; Monash University, Melbourne, Victoria, Australia

**Keywords:** *goal*, *hope*, *manipulation*, *patient decision-making*, *prospect theory*

## Abstract

It is widely supposed that it is important to ensure that patients undergoing medical procedures hope that their treatments will be successful. But why is hope so important, if indeed it is? After examining the answers currently on offer in the literature, we identify a hitherto unrecognized reason for supposing that it is important that patients possess hope for a successful treatment, which draws on prospect theory, Kahneman and Tversky’s hugely influential descriptive theory about decision-making in situations of risk and uncertainty. We also consider some concerns about patient consent and the potential manipulation of patients that are raised by our account.

## I. INTRODUCTION

It is widely supposed that it is important to ensure that patients undergoing medical procedures have hope that their treatment will be successful. Healthcare professionals often claim this, and bioethicists and philosophers who have commented on the issue usually seem to agree with them.^2^ But why is hope so important, if indeed it is? In this article, we identify what is (as far as we are aware) a hitherto unrecognized explanation for the importance of patients’ possession of hope for successful treatment, which appeals to prospect theory. This is Kahneman and Tversky’s (1979) hugely influential and well-replicated descriptive theory about decision-making in situations of risk and uncertainty.^3^ Because prospect theory is so influential, and because healthcare professionals and patients need to make decisions in situations of risk and uncertainty, there have already been many publications applying prospect theory to healthcare.^4^ Furthermore, it has been suggested that physicians should learn prospect theory.^5^ To our knowledge, however, no one has drawn the connection between hope in health care and prospect theory until now. By drawing out this connection, we provide new support for the widespread conviction that patient’s hopes matter, and we add to the ways in which prospect theory can be applied to decision-making in health care.

The article is organized as follows. In the remainder of this introduction, we say more about what hope is. In Section II, we look at current explanations for the importance of hope and show where and how our preferred approach relates to these. In Section III, we spell out relevant details of prospect theory, and we show how prospect theory helps account for the significance of hope in health care in Section IV. In Section V, we discuss some concerns about consent and the potential manipulation of patients that are raised by our account. A brief conclusion follows. Throughout the article, we work with the uncontroversial assumption that the healthcare professionals and bioethicists who regard patient hope as important also regard healthcare as an enterprise that is and should be dedicated to working with unwell patients to restore them to a state of good health. We agree with this assumption. Patients who are interested in pursuing some other end, such as the enhancement of their capacities over and above the levels required to enable good health, should seek assistance from some other sort of professionals than healthcare professionals.

Although we focus on hope in health care, we are aware that it is not only in health care that hope is regarded as important. It is often supposed that it is important that key military personnel are hopeful of success.^6^ It is also widely assumed that hope is crucial to elite athletic performance.^7^ We do not want to exclude the possibility that our analysis could be extended to these and perhaps other contexts in which hope is widely reckoned to be important. However, we do not attempt to extend it beyond the context of health care in this article.

There is sometimes said to be a “standard account” of hope in philosophy, which analyses hope as a belief–desire pair. Garrard and Wrigley characterize this account as follows: “A person hopes for a state of affairs, P, if and only if he desires that P, and believes that it is possible, though not certain, that P will come about” (2009, 39). One major concern with this standard account is that it fails to recognize that there is an affective dimension to hope. A patient might believe that there is a 1% chance that the malignant tumors will be successfully treated and approach the impending operation with a sense of hope of success in the face of great odds of failure. Or, a patient in similar circumstances, who believes that there is a 1% chance that her malignant tumors will be successfully treated may find herself in a state of despair, having abandoned hope in the face of great odds of failure. Both of these patients desire a particular state of affairs (that their malignant tumors are successfully treated) and believe that it is possible that the state of affairs will come about, so on the standard account, both possess hope. But, ordinary usage suggests that only one of them possesses hope. The other is in a state of despair, which is the very opposite of hope.^8^ We have more to say about despair and its relationship to hope in the next section of the article, as well as in Section IV.

Adrienne Martin influentially characterizes hope as involving an emotion that plays “a framing role in relation to our uptake, interpretation and deliberate use of information” (2008, 50). According to her, “to hope for an outcome is to *desire* (be attracted to) it, to assign a *probability* somewhere between 0 and 1 to it, and to judge that there are sufficient *reasons* to engage in certain feelings and activities directed toward it” (Martin, 2013, 7–8).^9^ Martin’s approach seems to capture something important about hope. Both of the patients in our example above react to the same information, but their emotional responses to that information are very different, and this difference appears to have important implications for their behavior. The patient who possesses hope of a cure is motivated to cooperate with healthcare professionals to attain a state of good health, whereas a patient who is in a state of despair is unmotivated to so cooperate. (In Sections II and IV, we discuss the motivating power of hope in more detail.)

In addition to contrasting her approach to hope to the “standard account” in philosophy, Martin (2008) contrasts her approach to one that is dominant in academic psychology and is particularly associated with the work of prominent “hope theorist” Charles Richard Snyder. Hope theorists in psychology have investigated links between hope and agency, and often stress the goal-oriented nature of hopes. We hope for a particular outcome, and being in a state of hope motivates us to seek the desired goal that is that outcome. Consistent with this way of thinking, Corn et al. define hope as a “goal-orientated cognitive construct with affective and behavioural implications” (2020, e452).^10^ The view we want to develop here is one that blends Martin’s approach with the approach to hope that has dominated academic psychology. As far as we can see, the two approaches are more compatible than Martin (2008) appreciates. The outcome Martin refers to in her aforementioned definition simply is the goal to which Corn, Feldman, and Wexler (2020) refer. We think that hope does have the framing role that Martin characterizes and crucially involves emotions. Also, the form of hope that we are interested in examining, the subset of patient hopes that align with the professional goals of the healthcare professionals attending to that patient, are goal-oriented. Healthcare professionals aim to restore patients to full health if possible. If a patient cannot be restored to full health, then healthcare professionals either aim to restore patients to an adequate but partial level of health, or broaden their aims beyond health, to include the goal of easing patient suffering, and, where patients are nearing the end of their lives, facilitating a good death for the patient.^11^ It could be pointed out, against Martin (2013) and Corn, Feldman, and Wexler (2020), that not all uses of the term “hope” seem to motivate goal-oriented behavior. A person might hope for pleasant weather next week, despite knowing full well that there is nothing that they can do to cause the weather to be pleasant next week. For this reason, we work with a modified version of Martin’s account of hope, according to which:

To hope for an outcome is to *desire* (be attracted to) it, to assign a *probability* somewhere between 0 and 1 to it, and, in situations in which it is possible to influence the probability of it occurring, to judge that there are sufficient *reasons* to engage in certain feelings and activities directed toward it.

Having introduced this complication, we set aside the issue of hopes that do not motivate goal-oriented behavior. It seems to us that patient hope for a cure is valued in health care because, *inter alia*, it motivates goal-oriented behavior: it motivates patients to cooperate with healthcare professionals to enable their own medical care and rehabilitation.^12^ As we demonstrate, this is not the only effect that hope has on patient behavior, but it is also one that needs to be considered.

## II. SOME CURRENT ACCOUNTS OF THE IMPORTANCE OF HOPE

One reason that is sometimes cited for the importance of hope refers to the psychological health benefits of having hope (Simpson, 2004). There may well be such benefits, but it is very difficult to tease them out from the psychological health benefits of optimism (Aspinwall and Leaf, 2002, 277).^13^ Whereas hope is always directed at a particular outcome, optimism is a tendency or disposition that need not be outcome oriented (Garrard and Wrigley, 2009, 39). Someone who is optimistic could be optimistic about the chances of a particular outcome occurring, or they might just be optimistic in outlook and disposed to see “the glass half full,” rather than “half empty.” Every instance of hoping involves a degree of optimism about an outcome occurring, but not every instance of being optimistic involves hoping, as not all instances of optimism are outcome oriented. Because hope is invariably accompanied by some optimism, it is hard to tell if there are psychological health benefits that are specific to hope and not to optimism. So, we doubt that pointing to psychological health benefits properly explains the stress placed specifically on patient hope in health care.

A second reason that is sometimes cited for the importance of hope is the importance of avoiding despair. Someone who falls into despair can end up lacking motivation to do anything. For example, Mason (2021) cites reports from Frankl (1946) of concentration camp inmates during the Second World War who fell into a state of deep despair about their miserable lives and would refuse to dress, or wash, or go out onto the parade grounds, even when guards threatened them and beat them. These inmates had become so despairing that they found themselves unmotivated even to try to avoid a bad death, resigning themselves to whatever treatment was meted out to them by the abusive guards who watched over them. Needless to say, they did not survive long. Frankl suggests that the need to avoid the dire consequences of despair underscores the importance of hope.^14^ We agree that despair is a terrible state to be in, but we do not regard despair as the mere absence of hope. There is a neutral state between hope and despair where one does not hope for a particular goal, but nor has one given up. Patients can be in a neutral state, neither hoping for the procedure they are undergoing to be successful, nor despairing that it is going to be unsuccessful, but simply going along with it. But, we take it that those who press the importance of patient hope in health care would not be satisfied with this neutral state. For this reason, we do not think that the appeal to the importance of avoiding despair is sufficient to explain the importance of hope in healthcare.

A third reason that is cited for the importance of hope in healthcare is one that has already been mentioned in passing, which is that hope motivates patients to cooperate with healthcare professionals to help achieve the goal of regaining a state of good health.^15^ In some cases, this is simply a matter of agreeing to be operated on and avoiding activities that might interfere with their recovery. In other cases, patients need to become and remain motivated to undertake regular exercises, stick to restrictive diets, and/or take regular medication for extended periods of time. We agree that motivation is a clear consequence of hope and is a reason why hope is important in health care. However, we do not think that this is the only reason why patient hope is important in healthcare. As we continue to argue, hope has a further consequence that often benefits patients, which is that it tends to change patient attitudes to risk, making them more willing than they would otherwise be to accept risks in order to try to attain states of good health. The argument for this conclusion and its importance is spelled out in Section IV of the paper. First, however, we need to describe the basic elements of prospect theory, which are drawn on in the argument to be spelled out in Section IV.^16^

## III. PROSPECT THEORY

To explain how prospect theory works, it helps to work with the example of Tversky and Kahneman’s (1981) much discussed “Asian disease problem.” Tversky and Kahneman’s research subjects were given the below passage:

Imagine that the U.S. is preparing for the outbreak of an unusual Asian disease, which is expected to kill 600 people. Two alternative programs to combat the disease have been proposed. Assume that the exact scientific estimates of the consequences of the programs are as follows:

If Program A is adopted, 200 people will be saved.

If Program B is adopted, there is a one-third probability that 600 people will be saved and a two-thirds probability that no people will be saved. (1981, 453)

They were then asked to indicate a preference between the two programs. 72% preferred Program A and 28% preferred Program B.

Another group of research subjects was presented with the same scenario, and then asked to indicate a preference between two further programs to combat the unusual Asian disease, Program C and Program D. These research subjects are informed that:

If Program C is adopted, 400 people will die.

If Program D is adopted, there is a one-third probability that nobody will die and a two-thirds probability that 600 people will die. (Tversky and Kahneman, 1981, 453).

This time, 22% of subjects preferred Program C and 78% preferred Program D.

These results are striking because all four options have the same expected utility, Program C is just Program A “reframed,” and Program D is just Program B reframed. The reframing involved is more specific than the framing that was discussed earlier when Martin’s characterization of hope was under consideration. In this case, a seemingly minor shift in the language used to describe a pair of scenarios has occurred and caused a major shift in the preferences of research subjects. In the “lives saved” frame, the overwhelming majority of research subjects expressed risk-averse preferences. They preferred the sure bet of saving 200 lives to the risks involved in trying to save all lives. However, in the “lives lost” frame, the overwhelming majority exhibited risk-seeking preferences. They were willing to risk all 600 lives to try to save everyone, even though they were offered the risk-averse option of limiting deaths to 400. How can we explain this dramatic shift?

According to prospect theorists, the aforementioned shift in preferences occurs because of a change in “reference point.” This is the point as which we, consciously or unconsciously, distinguish gains from losses. In the lives saved frame, it is implicitly assumed by most research subjects that the lives that may be saved will otherwise die, so every outcome other than all people dying is construed as a gain. However, in the lives lost frame, it is implicitly assumed by most subjects that the lives that are in danger of being lost will otherwise survive, so every outcome other than all people living is construed as a loss. The linguistic shift between the lives saved frame and the lives lost frame shifts our implicit neutral reference point and so shifts our understanding of what counts as a gain and what counts as a loss. The shift from treating an outcome as a gain and treating an outcome as a loss might not seem important, but prospect theorists have assembled a large body of evidence indicating that it is psychologically important. It seems to be an ingrained feature of ordinary human psychology that we tend to adopt a risk-averse attitude to perceived gains and a risk-seeking attitude to perceived losses.^17^

Prospect theory involves three core claims.^18^

1. We evaluate risks in comparison to a “reference point.” Outcomes understood as being superior to this reference point are regarded as gains. Outcomes understood as being inferior to this reference point are regarded as losses.
2. We are loss averse. Losing hurts us psychologically more than winning benefits us psychologically.
3. We experience diminishing sensitivity to gains and losses in proportion to their relative distance from a reference point. If our reference point is $0, then the first $10 we might gain, or might lose, has more psychological significance for us than the next $10 we might gain, or might lose, and so on.

**Fig. 1.**
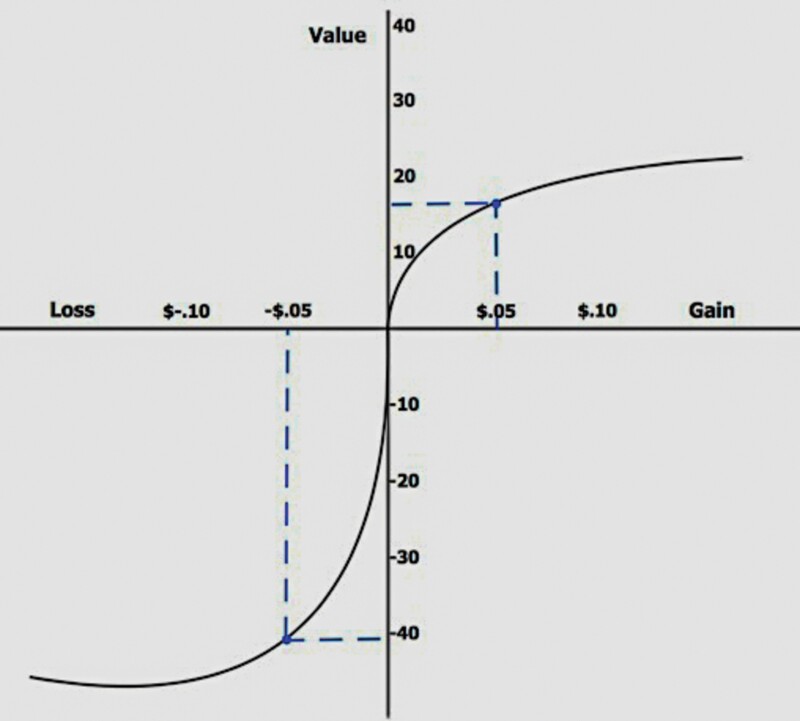
Outcomes of prospect theory.

Together, the three core principles of prospect theory give us outcomes that can be illustrated as per Fig. 1.^19^

For many of the ordinary decisions that we make, our reference point is the status quo. If a person currently owns exactly one house—their personal status quo—and they are threatened with repossession due to accumulated debt, they will take more risks to protect that house from repossession than they will if they are presented with an opportunity to borrow money and purchase a second property. They are risk averse in regard to risky opportunities that may take them above their reference point, and risk-seeking in regard to threats to take them below their reference point. This reference point could shift if the person in question acquired reason to expect that they will soon come into possession of a second property—perhaps as a result of an inheritance. If they regard the soon-to-be-inherited property as being as good as theirs already, then they will take more risks to protect their anticipated inheritance from legal challenge than they would if they merely regarded the inheritance as a lucky windfall that they might possibly come to possess at some point. What drives their increased appetite for risk is a shift in reference points brought on by their reason-based expectations.

As well as setting reasonable expectations of gain (or loss), or using language that leads people to regard outcomes that would otherwise be regarded as losses as gains (or vice-versa), as in the Asian disease study, there are other ways to shift a person’s reference points. One well-established means of shifting a reference point is by setting a goal (Kahneman, 2011, 302–4). When persons adopt a goal, it becomes their reference point. They come to regard a result that falls short of a goal as a loss, and a result that meets the goal or exceeds it as a gain. A runner who sets themselves a goal of running 5km will regard an inability to make that distance, perhaps because of leg soreness, or a stitch, as failure. They will try harder and take more risks with their health to meet that goal than they will to exceed it. Once they have met their goal, their behavior is likely to shift from risk-seeking to risk averse. They are much less likely to risk their health to exceed a goal than to risk their health to meet that goal.

Pope and Schweitzer (2011) demonstrated the capacity of goals to serve as reference points by examining 2.5 million golf putts using laser measurements. They reasoned that if prospect theory is correct and goals serve as reference points, then professional golfers would treat a par score as a reference point on most holes, on most courses, because a par round (of 18 holes) is what competent professional golfers are usually expected to aim for and achieve. If professional golfers treat a par score as a reference point, then they can be expected to take more risks to avoid an above-par score (a bad outcome in golf) than they would to achieve an under-par score (a desirable outcome in golf). Pope and Schweitzer’s (2011) study confirmed this hypothesis. They found that when putting for birdie (one under par), professional golfers tend to hit the ball less hard (and sometimes insufficiently hard to land the ball in the hole) than they do when putting for par. Pope and Schweitzer (2011) propose that this difference is consistent with, and is plausibly explained by, prospect theory. When putting for birdie, golfers likely consider that they are “ahead,” having gained something (needing fewer shots on this hole so far than par), and so become risk averse about their putt possibly missing or overshooting the hole, to protect their lead (gain), and thus tend not to hit the ball very hard (and sometimes less hard than is necessary to land the ball in the hole). Pope and Schweitzer comment that “players sacrifice success when putting for birdie to avoid difficult follow-up putts” (2011, 131). However, when putting for par, golfers likely consider that they have gained nothing (they are not ahead but are simply on par) and stand to lose something, and so become risk-seeking about their putt possibly missing the hole and resulting in an above-par score (loss), and thus tend to hit the ball harder (and those putts actually end up landing the ball in the hole more often than do birdie putts).^20^

## IV. PROSPECT THEORY APPLIED TO PATIENT DECISION-MAKING

Does prospect theory describe patient decision-making with respect to health better than rival accounts of decision-making under risk, such as expected utility theory? It would be surprising if it did not, given the strong evidential base for the descriptive accuracy of prospect theory,^21^ and an absence of reasons to suggest that people approach decision-making under risk when it comes to making decisions about health in a markedly different way from decision-making under risk when making decisions about other topics. Furthermore, the evidence we have suggests that patients often make decisions about risks to their future life expectancy in ways that are well described by prospect theory.^22^ What the aforementioned studies all suggest is that patients make decisions about their future life expectancy in relation to a reference point, with risks regarding potential losses to life expectancy looming larger in their thinking than potential opportunities to improve their life expectancy.

In the absence of an intervention that can shift an unwell patient’s reference point, with respect to their life expectancy, or other aspects of their health, their reference point can reasonably be expected to be their current state of health—the status quo (Treadwell and Lenert, 1999, 345).^23^ This patient will be risk-seeking when making attempts to prevent further *deterioration* of their health, and risk averse with respect to attempts to *improve* their health from its current state. Unfortunately, a risk-averse attitude can sometimes be a barrier to the successful treatment of health conditions. A risk-averse patient may be unwilling to consent to treatment plans that involve the risk of significant side effects. Furthermore, the risk-averse patient who has been treated may lack sufficient motivation to take risks involved in rehabilitation plans. They are less likely to take medicines that involve risks of side effects, as part of their rehabilitation, and less likely to perform exercise that involves the risk of injuries, as part of their rehabilitation.

The realization that people’s decision-making about their health is significantly affected by the location of their reference point provides an opportunity to shape patient decision-making by making interventions that serve to shift a patient’s reference point (Schwartz, Goldberg, and Hazen, 2008). One way to do this is for healthcare professionals to persuade a patient to adopt a particular goal, such as the goal of returning to a former state of health. If unwell patients take on the goal of returning to their former state of health, then their reference point shifts, and they will tend to be risk-seeking in regard to any outcome that falls short of that goal. They will be likely to be more willing to accept the risks of side effects when undergoing operations, taking medication, and undertaking exercises as part of rehabilitation programs. They will be likely to become risk averse only after they have retained a former state of health and are contemplating the possibility of improving their state of health above what they enjoyed in their former state of health. When a healthcare professional succeeds in imbuing patients with the hope of regaining a previous state of health, the patients take on the goal of cooperating with healthcare professionals to improve their health until it is returned to that former state.

The close relationship between goals and hopes, recognized by Snyder, Rand, and Sigmon (2005) is, we think, key to understanding the importance of hope in health care. When a patient embraces a particular hope, they also embrace the goal of realizing that hope, and their adoption of this goal shifts their reference point, changing their attitude to risk. The motivational effects of hope, recognized by Garrard and Wrigley (2009), are important as well. Recovering patients need to remain motivated to complete courses of rehabilitation involving exercise, dietary restrictions, and/or medication. Some of these courses of rehabilitation can go on for months or even years, and hope of recovery is crucial in enabling patients to remain motivated to complete such programs.

As well as using prospect theory to understand the importance of hope in health care, it can be used to shed light on the difference between the absence of hope and despair, which was mentioned in Section II. A patient who have no particular hope of recovery (though recovery is nevertheless *possible* for them) and is perhaps experiencing sadness and grief at their imminent mortality without being in a state of despair, has a reference point at the status quo—their current state of health. They will tend to be risk averse with respect to proposed treatments to improve their health, and will tend to have a risk-seeking response to threats that their health will further deteriorate. A patient who is in a state of despair has, in effect, accepted that their health will further deteriorate. Their reference point is the state of affairs that, they assume, their health will reach after deterioration ceases. If they have a life-threatening condition, then they may assume that they will die and they will likely regard themselves as being “as good as dead” already. This imagined future state serves as their reference point. They will tend to be risk averse with response to proposed interventions to prevent them from declining to that imagined future state, and unmotivated to cooperate with any efforts made by healthcare professionals to improve their health, or even to prevent further deterioration in their health. Some patients who are despairing about the possibility of recovery and accept that they are “as good as dead” will, nevertheless, go on to acquire a new goal which is to experience as good a death as possible.^24^ The details of individual conceptions of a good death vary, but in many cases, it involves aiming to experience a death that is as pain-free as possible. However, a patient who is in a state of deep despair may, as we saw with Frankl’s example of despairing concentration camp inmates, find themselves psychologically unable to acquire new goals, and will be unwilling to take risks even to secure the goal of avoiding a bad death, simply abandoning themselves to whatever outcome with which fate provides them.

## V. HOPE, MANIPULATION, AND RISKY MEDICAL PROCEDURES

Because patients’ hopes can be a powerful force and can have significant effects, healthcare professionals should be mindful about the potential impacts on hope of their communications with patients. When a healthcare professional instills hope in a patient, the healthcare professional induces the patient to take on a new goal. And (as we explained above), when considering their options, patients will often use that goal as their reference point, and be willing to take greater risks to achieve that goal.^25^ One concern is the problem of deceptively imbuing a patient with “false hope.” There is a sizeable literature on false hope in health care, much of it devoted to working out what counts as false hope, and working out if, when, and why instilling false hope in a patient is morally problematic.^26^ We follow Musschenga (2019) in understanding false hope as epistemically unjustified hope, and in holding that it is morally wrong to cause a patient to have false hope.

Concerns about deceptively imbued false hope do not exhaust the range of concerns that arise when we consider the situation of a healthcare professional attempting to instill hope in a patient. Because hope can change a patient’s attitudes to risk, it raises concerns about patients being manipulated into accepting risks involved in medical procedures that they might not otherwise accept—and (in one important class of cases of wrongful manipulation of patients by doctors) being led by *means* that they disapprove of, to accept those risks. And, as we will see, these concerns can arise even when patient hopes are epistemically justified.^27^ How are we to respond to these concerns?

Let us consider the aforementioned concerns in the context of discussions between doctors and patients with advanced cancer. A doctor might instill hope in such patients to induce them to become more risk-seeking (and so less risk averse) in their cancer treatment decisions. For example, suppose a patient has advanced cancer which has proven resistant to safe, standard treatments, and all that remains that she is aware of are drugs which are very unlikely to cure her cancer and are highly likely to cause her premature death. If this patient no longer has hope that there will be a cure for her cancer, she will likely be risk averse in regard to such drugs, and so unwilling to try them. But, suppose that the doctor becomes aware of another drug that has been approved for use in treating this form of cancer, though only for patients in age groups slightly younger than this patient. The doctor may be willing to provide this patient with an “off-label” prescription^28^ for this drug, on the basis of reasonable quality evidence both that the drug can effectively treat this cancer in slightly older patients than those for whom it has been approved, and that the drug is far less likely to cause the premature death of patients in her age group than are the other drugs of which this patient has been made aware. Yet, the patient remains unhopeful of a cure and so her risk aversion leads her to decline the doctor’s prescription. If, however, the doctor provides her with grounds for hope that this drug can successfully treat her cancer (telling her about the evidence for the effectiveness of this drug in younger patients, and about the evident risks of this drug), and the patient acquires hope that her cancer can be successfully treated, the patient thereby acquires the goal of cooperating with her doctor to enable her cancer to be successfully treated. In acquiring this new goal, the patient shifts her reference point, and is likely to become more risk-seeking in the pursuit of that goal—because she would come to regard falling *short* of that goal as a *loss*. On our account, this is due to the patient shifting her reference point from one where she did not have hope for a cure (and so her reference point was her current state, leading her to be risk averse regarding drugs that might prevent her from declining further), to a reference point of regarding a full recovery as now *achievable*, and so she becomes risk-seeking, in pursuit of that goal.

As far as we can see, the doctor in the above example has influenced the patient’s decision-making by providing her with grounds for hope, thereby provoking her attitude to risk to shift in ways that she may not be consciously aware of, but the doctor has not done so in a way that is morally unacceptable. The doctor has fulfilled the ordinary obligations of the informed consent process and assisted the patient in making her own decision about whether or not to take the new drug. The patient has become hopeful after listening to the doctor, and this has led to a shift in her attitude to risk, raising concerns about the possibility of manipulation. But we think these concerns are mitigated by an appreciation of prospect theory. What is important to understand is that the doctor has not deceptively induced the patient to take on an attitude to risk that she does not endorse, nor has the doctor induced her to take on an attitude to risk that is irrational. The patient, like everybody else, according to prospect theory, is both a risk seeker and risk averse, and switches between the two states on a regular basis. When she makes a decision after switching between the two states, on the basis of acquiring hope, she is making that decision in a way that remains authentically her own. And, it is not irrational to switch from risk aversion to risk-seeking on the basis of acquiring hope. Being risk-seeking can be inappropriate in some circumstances, but it is not an irrational state of mind. The same can be said for being risk averse.

However, if a doctor knowingly attempts to instill hope in a patient by targeting a decision-making weakness^29^ which the patient prefers not to influence their decisions, and the patient is thereby led to become risk-seeking due to their newfound hope shifting their goal and hence their reference point, the doctor is unjustifiably manipulating this patient. Joel Rudinow argues that “A attempts to manipulate S if and only if A attempts the complex motivation of S’s behavior by means of deception or by playing on a supposed weakness of S” (1978, 346). What Rudinow means by the “complex motivation” of someone’s behavior is, attempting to “motivate someone’s behaviour in a way which one presumes will alter (usually by complicating) the person’s project (complex of goals)” (1978, 345).^30^ Consider an example where a cancer patient has decided to forego a third round of chemotherapy, because the evidence indicates that it is ineffective against this form of cancer, and carries a risk of causing premature death, and no other effective intervention seems to be available. Suppose that this patient has a tendency to “clutch at straws” when hearing about procedures that could be interpreted as offering even a slim chance of benefiting them, and that the patient regards this tendency as a decision-making weakness, preferring overall not to be so inclined.

Now suppose that the patient’s oncologist has a single-minded focus on preventing patients’ deaths at all costs, and (knowing of their patient’s tendency to clutch at straws) stresses the evidence of efficaciousness found in a new trial of three rounds of chemotherapy, and instills enough hope to shift their patient’s reference point, such that the patient now regards themselves as having failed if they do not agree to another round of chemotherapy. That is, the patient now regards the state of being unwell with cancer as a *loss*, whereas, prior to investing their hopes in a third round of chemotherapy, the patient had been reconciled to (accepting of) the idea that declining another round of chemotherapy involves no loss (of anything of future significance) to them. If this patient is thereby led to become more risk-seeking, in agreeing to undertake a risky third round of chemotherapy, then this patient has arguably been manipulated (and wrongfully so) by the oncologist despite not having been deceived in any way by that oncologist.^31^

A key ethically concerning feature of such cases is *how* patients are induced to become more risk-seeking—that is, through the targeting of a decision-making weakness of theirs (their tendency to clutch at straws) which they strongly prefer *not* to influence what they regard as achievable—and what they decide to undertake, in light of now regarding a particular outcome as more achievable than it actually is.^32^ When a patient who values deciding between healthcare options on the basis of an accurate understanding of the risks and benefits is imbued with hope by a doctor, in order to induce the patient to become more risk-seeking, they are led to do so by acquiring a new goal and reference point. And, when the doctor targets what this patient regards as a decision-making weakness, by using hope to induce the patient to acquire a new goal in a way that bypasses rational deliberation about risks, this doctor is arguably manipulating the patient in ways which wrongfully compromise this patient’s autonomy.^33^

## VI. CONCLUSION

We have argued that a key reason for the importance of patient hope in health care is that hope has the capacity to set goals, thus shifting reference points and thereby changing patient attitudes to risk. In developing this argument, we have drawn on prospect theory and demonstrated how this theory helps account for the significance of hope in patient decision-making in situations of risk and uncertainty. We pointed out that the ability of healthcare professionals to influence patient hopes and thereby change their decisions, by changing their attitudes to risk, raises important concerns about the possibility of patients being manipulated into accepting risks involved in medical procedures that they might not otherwise accept.

Despite these concerns, there are many situations in which it is perfectly appropriate for healthcare professionals to imbue patients with hope, as we show.

## Footnotes

2 See, for example, Musschenga (2019), Eaves, Nichter, and Rittenbaugh (2016), McMillan, Walker, and Hope (2014), Sharot, Korn, and Dolan (2011), Garrard and Wrigley (2009), Hagerty et al. (2005), Simpson (2004), and Pellegrino, Thomasma, and Miller (1996).

3 In a major recent study, Ruggeri et al. (2020) replicate the descriptive accuracy of prospect theory for financial choices among cohorts of experimental subjects in 19 countries.

4 See, for example, Gisbert-Perez, Marti-Vilar, and Gonzalez-Sala (2022), Attema et al. (2019), Focella, Zikmund-Fisher, and Shaffer (2016), Schwartz, Goldberg, and Hazen (2008), van Osch, van den Hout, and Stiggelbout (2006), Moffet and Suarez-Almazor (2005), Rivers et al. (2005), Winter, Lawton, and Ruckdeschel (2003), and Treadwell and Lenert (1999).

5 See Verma, Razak, and Detsky (2014).

6 See, for example, Matthews et al. (2006).

7 See Curry and Shyder (2000).

8 It is important to acknowledge that although many people experience feelings of despair when they come to accept that their death is near, there are some who do not experience despair when faced with the prospect of impending death. One can imagine devout Christians who were convinced that they were soon to enter Heaven looking forward to death. A range of other emotional responses to the prospect of death is also possible.

9 Note that Martin’s account of hope does not involve any assumption that the judgment that there are sufficient reasons present is itself a rational judgment. A judgment regarding the presence of sufficient reasons to engage in particular feelings and activities can either be a rational or an irrational judgment.

10 See also, Snyder, Rand, and Sigmon (2005).

11 In doing so, healthcare professionals ought to take into account what the patient regards as a good death.

12 Note that an individual’s hope can also motivate them to pursue their own personal goals. For example, a patient’s hope for a good death can motivate them to pursue this, whether or not a healthcare professional regards facilitating a good death for that patient as one of their professional goals.

13 For general discussion of the psychological benefits of optimism, see Seligman (2006). For discussion of the health benefits of optimism to (particular types of) patients, see He et al. (2014), Marton et al. (2022), and Scheier et al. (1989).

14 See the discussion of Frankl’s views in Mason (2021).

15 As we noted earlier, an individual’s hope can also motivate them to pursue their own personal goals (such as the goal of a good death), whether or not an individual’s personal goals are consistent with clinical goals.

16 We do not take our discussion of the importance of hope in health care in this section to be exhaustive. Our primary concern is to identify a hitherto unrecognized reason why hope is important in health care and not to provide a comprehensive discussion of all reasons for regarding hope in health care as important. One reason for doubting that it is exhaustive is that our focus is on current philosophical, psychological, and bioethical accounts of the importance of hope in health care. However, there is conceptual work on hope in health care from a range of disciplines that would need to be considered in a comprehensive treatment of hope in health care. These disciplines include anthropology (e.g., Kaptchuck et al., 2009), sociology (e.g., Petersen and Wilkinson, 2015), and theology (e.g., Moltman, 2012).

17 For a general discussion of prospect theory and the evidence for it, see Kahneman (2011).

18 For a more detailed description of the three central claims of prospect theory, see Kahneman (2011).

19 See also Kahneman (2011).

20 Interestingly, some professional golfers, including Tiger Woods, demonstrate an awareness of the psychological difference in their play when putting for birdie and putting for par. Pope and Schweitzer quote Woods as saying “Any time you make big par putts, I think it’s more important to make those than birdie putts. I don’t ever want to drop a shot. The psychological difference between dropping shot and making a birdie, I just think it’s bigger to make a par putt” (2011, 144–5).

21 See Kahneman (2011), Ruggeri et al. (2020).

22 See Winter, Lawton, and Ruckdeschel (2003), Moffett and Suarez-Almazor (2005), and van Osch, van den Hout, and Stiggelbout (2006).

23 A complicating factor is that, as health economists recognize, health is always on a slow decline as our bodies age and we slowly expend our remaining supply of “quality adjusted life years (QALYs).” However, people do not usually appear to take this fact into account when they think about their health. This is, in part, because they do not usually know how long they have to live, and so, how many QALY’s they have remaining, and in part because we usually assess our future health prospects in terms of gains and losses from a fixed reference point, rather than from a shifting reference point. (See Busschbach, Hessing, and de Charro, 1993; Eisenberg et al., 2011; and Mitnitski et al., 2022). We thank an anonymous reviewer for this point.

24 Thanks to an anonymous reviewer for help here.

25 We are not here suggesting that this occurs in *every* case where hope is instilled in a patient.

26 See, for example, Simpson (2004), Musschenga (2019), and Hordijk, Vermuelen, and Bunnik (2022).

27 We here adopt Musschenga’s account of justified hope: “Hope is justified when the hoping person knows and accepts experts’ judgement about the probability of hope fulfilment” (2019, 432).

28 “Off-label prescribing is when a physician gives you a drug that the U.S. Food and Drug Administration (FDA) has approved to treat a condition different than your condition” (https://www.ahrq.gov/patients-consumers/patient-involvement/off-label-drug-usage.html). “Off-label use also includes prescribing a drug for a different population or age range than that in which it was clinically tested and using a different dosage or dosage form” (Furey and Wilkins, 2016, 588). There can be robust evidence in support of off-label prescribing in particular cases—see, for example, the advice given to doctors by the National Prescribing Service in Australia: “If, in the opinion of the prescriber, the off-label prescription can be supported by reasonable quality evidence, for example, the indication is identified in the Australian Medicines Handbook, the prescriber should proceed if this is in the patient’s best interests” (https://www.nps.org.au/australian-prescriber/articles/off-label-prescribing). For a helpful discussion of the ethics of off-label prescribing, see Largent, Miller, and Pearson (2009).

29 An example of a “decision-making weakness” that we go on to discuss is having a tendency to “clutch at straws.”

30 As Noggle (2022) indicates, this particular disjunctive analysis of manipulation has much to recommend it, given that manipulation seems to be a rather heterogeneous concept. Also, Rudinow’s emphasis on decision-making weakness in the target of manipulation attempts also coheres well with an influential unified account of manipulation developed by Noggle (2020).

31 Note that we are not claiming that every cancer patient who is induced by their oncologist’s highlighted reports of new experimental findings to start hoping that a further round of chemotherapy might cure them—and to thereby shift from regarding declining another round of chemotherapy as no loss (of anything of future significance to them) to regarding declining this as a loss—will then undergo another round of chemotherapy. Deciding about such a course of action would commonly involve deliberating about further considerations, such as burdens imposed on their carers, etc. We are not claiming that every time a newfound hope shifts one’s reference point that this will always be a *decisive* consideration for the agent.

32
Todd (2013) argues that ethically problematic manipulation of one person by another typically involves having an understanding of *specific* decision-making weakness/es in the target (whether or not the manipulator also has an understanding of decision-making weaknesses in human beings *generally*). See also Grant (2011).

33 See also Cocking and Oakley’s (1994) discussion of concerns about researchers manipulating patients into joining clinical trials by framing risk information in particular ways.
